# Pain interference type and level guide the assessment process in chronic pain: Categorizing pain patients entering tertiary pain treatment with the Brief Pain Inventory

**DOI:** 10.1371/journal.pone.0221437

**Published:** 2019-08-20

**Authors:** Teemu Miettinen, Hannu Kautiainen, Pekka Mäntyselkä, Steven J. Linton, Eija Kalso

**Affiliations:** 1 Division of Pain Medicine, Department of Anesthesiology, Intensive Care and Pain Medicine, University of Helsinki and Helsinki University Hospital, Helsinki, Finland; 2 Folkhälsan Research Center, Helsinki, Finland; 3 Institute of Public Health and Clinical Nutrition, University of Eastern Finland, Kuopio, Finland; 4 Primary Health Care Unit, Kuopio University Hospital, Kuopio, Finland; 5 Department of Law, Psychology and Social Work, Center for Health and Medical Psychology, Örebro University, Örebro, Sweden; Monash University, AUSTRALIA

## Abstract

Chronic pain patients enter treatment with different problem profiles making careful assessment a necessity for more individualized treatment plans. In this cross-sectional study we assigned 320 patients entering tertiary multidisciplinary pain treatment into four categories based on whether they scored low or high on the activity and the affective pain interference dimensions of the Brief Pain Inventory (BPI). To determine whether this categorization system delineates issues that should be assessed further, the categories were compared with ANOVA and MANOVA analyses on three domains: variables affecting physical well-being (body mass index, exercise, substance use), psychological resources (mood), and pain-specific psychological factors (pain anxiety, pain acceptance). The results indicated that subjects who scored low on both interference dimensions compared similarly in weight: mean Body Mass Index (BMI) 27.0 (SD 6.0) kg/m^2^, and exercise: mean of 2.4 (SD 1.7) exercising sessions over 20 minutes per week, to the general population, had no depressive symptoms on average: mean Beck Depression Index II (BDI-II) score 11.7 (SD 7.5), and had the most favorable psychological reactions to pain relative to the other categories: mean total Pain Anxiety Symptoms Scale-20 (PASS-20) score 36.4 (SD 17.9). In contrast, when interference was high on activity, more physical well-being problems were evident e.g. weight: mean BMI 31.0 (SD 7.3) kg/m^2^, diminished exercise: mean of 1.5 (SD 1.6) exercising sessions per week, and avoidance behavior: mean PASS-20 Escape/Avoidance subscale 3.7 (95% CI: 1.7 to 5.8) scores higher in comparison to activity interference remaining low. With high affective interference, more depressive symptoms: mean BDI-II score 17.7 (SD 7.3), and more cognitive pain anxiety: mean PASS-20 Cognitive Anxiety subscale 2.8 (95% CI 0.7 to 4.8) scores higher in comparison to affective interference remaining low, emerged. Having high interference on both dimensions indicated accumulated risks for reduced physical well-being: mean BMI 29.9 (SD 6.1) kg/m^2^, mean of 1.2 (SD 1.7) exercising sessions per week, mood problems: mean BDI-II 20.3 (SD 10.6), and negative psychological reactions to pain: mean total PASS-20 score 53.2 (18.4). The results suggest that low interference on both dimensions may allow assessment with only physician consultations, while high interference on either dimension may call attention to distinct issues to be addressed with the help of a physiotherapist or a psychologist, whereas high interference on both dimensions highlights the need for a full multidisciplinary assessment.

## Introduction

Multidisciplinary pain treatment aims to address the biological, psychological, and social factors contributing to the chronic pain problem [1]. Patients present with varying degrees of complexity within these factors and to help the patient receive the best treatment, a multiprofessional team including a variety of professions e.g. a physician, a physiotherapist, and a psychologist perform the assessment. Information on the diagnosis and of the intensity of pain is not enough for understanding what components of multidisciplinary treatment the patient may need. The purpose of this study was to explore whether classifying patients on pain interference, which refers to the impact that pain has on the patient’s life activities, may help to streamline the assessment procedure.

The Brief Pain Inventory (BPI) [2] is a widely used basic questionnaire assessing pain interference. The developers of BPI propose that pain interference comprises two dimensions: “activity interference” (including walking, work, general activity), and “affective interference” (including mood, relation with others, enjoyment of life, sleep) [3]. Many interference studies combine the measured areas into a single index, but having two dimensions may have benefits. First, the two dimensions may be related to different problems that need to be addressed in the treatment. Affective interference has been associated more with pain catastrophizing, depression [4], and pain intensity [5] than activity interference. Second, the two dimensions may be equally or unequally affected, e.g. one being high and the other low, and this information disappears if the items are reduced to a single index.

The study analyses whether categorizing pain patients by activity and affective interference will reveal the issues that may appear in the treatment regarding the three domains: risks for physical well-being (exercise, body mass index, and substance use), psychological resources (mood), and pain-specific psychological factors (pain anxiety and pain acceptance). Should categories with different problems appear, the findings may help in choosing the areas that require further assessment.

Previous research has linked higher pain interference to less recreational exercise [6], weight gain in women [7], and nicotine and alcohol dependence [8]. These factors may impede pain management when poor physical condition makes physical rehabilitation more demanding for the patient [9], when obesity causes the pain to spread to areas beyond the original ones [10], or when substance use maintains passive coping with pain or anxiety [11,12].

In both prospective [13,14] and cross-sectional studies [15], higher pain interference has been associated with more depression or anxiety symptoms. Depression and anxiety bring forth negative emotional states that may intensify the unpleasantness of pain [16], reduce the patient’s psychological resources, and diminish treatment results [17].

Patients with less pain catastrophizing and more self-efficacy [18] or better pain acceptance [19,20] have reported less pain interference. Thus, helping the patient to reduce pain-related anxiety or to adopt a more accepting attitude towards pain might help in reducing the impact of pain.

In this study, we categorize pain patients to those who are low on both activity and affective interference, to those with high interference on either dimension, and to those with high interference on both dimensions at the same time. Our hypothesis is that high activity interference is associated with less exercise [6], and high affective interference is associated with more depressive symptoms and pain-related anxiety [4]. How high interference that is limited to one dimension differs from high interference across both dimensions is a novel question.

## Material and methods

### Design

This study employs a cross-sectional design. Patients seeking care for a persistent pain problem completed the Brief Pain Inventory and were then classified according to their scores on both activity and affective pain interference to either a “low” (under 7.00) or “high” level (equal or above 7.00) [18,21,22]. This produced four distinct groups: A (low activity interference/high affective interference), B (high activity interference/high affective interference), C (low activity interference/low affective interference), and D (high activity interference/low affective interference). The groups were then inspected for differences on variables affecting physical well-being, psychological resources, and pain-specific factors.

### Subjects

The cohort comprised 320 patients participating in the KROKIETA study, which explores relationships between socioeconomic factors, lifestyle aspects, biochemical variables, psychological factors, and quality of life of patients with chronic non-malignant pain [23]. The KROKIETA study was conducted as a multicenter study in three multidisciplinary pain clinics and three facial pain clinics in Finland. The patients attending the facial pain clinics were excluded from the current study, as only a part of the measures was collected from them. The coordinating ethics committee of the Helsinki and Uusimaa Hospital District approved the study protocol (29/13/03/00/12). All participants provided written informed consent.

Patients received an invitation to the KROKIETA study before their first visit to the pain clinic by a leaflet included in the mailing containing the pre-appointment questionnaires on pain, health, and lifestyle factors. If the patient agreed to participate in the study, he/she signed the informed consent form at the clinic and filled in further questionnaires on psychological factors and quality of life, which were then input into the study database along with data from the pre-appointment questionnaires. During the visit a nurse measured the patient’s weight, height, and blood pressure, while the physician recorded information on diagnoses, medications, and treatments. Approximately half of the invited patients declined participation in the study.

### Measures

#### Brief pain inventory

Brief Pain Inventory [2] is a questionnaire designed to capture both pain intensity and the amount of interference that pain has on functioning. BPI has been extensively used in both cancer- and non-cancer-related pain. Pain intensity is measured with four items (worst, least, on average, and currently). Interference is measured with seven items, including general activity, mood, walking, work (including paid and household work), relations with others, sleep, and enjoyment of life. The patient answers the items on a scale of 0–10, the highest number indicating the worst imaginable pain for intensity items and complete interference for interference items.

Several reports have presented a proposed factor structure with pain intensity, activity interference, and affective interference factors [3,24,25], and internal consistencies have been good for the scales (α = .87, .92, and .91, respectively) [26]. Walton et al. [4] confirmed the two main interference factors, but had a sleep item as a third independent factor. Some other researchers have ended up with a one-factor solution on interference items [27–29].

For this study, we used the mean score of the pain intensity items and constructed the interference scales as proposed by Cleeland et al. [3]: activity interference (walking, work, general activity) and affective interference (mood, relation with others, enjoyment of life, sleep).

#### Variables affecting physical well-being

Body Mass Index (BMI) was calculated as an indicator for overweight (>25.0 kg/m^2^) and obesity (>30.0 kg/m^2^) from measurements taken by the nurse during the first visit at the clinic.

Exercising activity was assessed with a question asking the subject to estimate the weekly frequency of free-time physical exercise producing at least slight shortness of breath and perspiration and lasting for a minimum of 20 minutes at a time. The subject could also indicate if no exercise was possible for health reasons. The prospective nationwide health study FINRISK has used this measure for over 30 years [30]. Increasing self-reported exercise frequency has been associated with increasing maximal oxygen consumption measured in the treadmill test and activity category measured by an electronic activity monitor [31], and increasing self-perceived fitness [32].

Smoking status was assessed with two questions from the FINRISK study [30]. A current smoker was defined as having smoked on a regular basis for at least one year and continuing to smoke.

Alcohol Use Disorders Identification Test (AUDIT) [33] was used to evaluate alcohol risk use. AUDIT is sensitive to current problematic drinking as opposed to previous alcohol abuse. AUDIT consists of ten questions, with each response scored from 0 to 4. A cut-off value of 8 indicates problematic drinking [34]. Internal consistency for the scale has been good across several studies (median α = .83), and it has worked accurately as an alcohol screen among various types of subjects and settings [35].

#### Mood

Beck Depression Inventory-II (BDI-II) [36] was used to assess depressive mood. BDI-II is a commonly used questionnaire to measure depressive symptoms for both clinical and research purposes. It includes 21 items, with each item yielding a score between 0 and 3. The item sum score is interpreted as reflecting either mild (14–19), moderate (20–28), or severe (>28) depressive symptomatology. Psychometric properties of BDI-II have been established among chronic pain patients, with excellent internal consistency for the scale (α = .92) [37].

#### Pain-related anxiety

Pain Anxiety Symptoms Scale is a measurement assessing pain-related anxiety. In this study, we used the 20-item version (PASS-20) [38]. PASS-20 has four anxiety subscales: cognitive anxiety, escape/avoidance behavior, fear of pain, and physiological anxiety symptoms. The subject answers the items on a 6-point scale ranging from “never” to “always”. Internal consistencies for the scales have been acceptable (α = .86 for cognitive, α = .75 for escape/avoidance, α = .82 for fear, and α = .81 for physiological anxiety) [38], and studies have confirmed the factor structure in low back pain, fibromyalgia, and non-clinical samples [39,40].

There are no established cut-off values for what constitutes an abnormal anxiety score in PASS-20. Abrams et al. [40] proposed a total score of 30 to indicate a high risk for developing problematic pain-related anxiety among acute pain patients presenting in primary care, also noting that the score may be overly cautious. Categories of mild (total score < 34), moderate (34–67), and severe pain anxiety (> 67) were used in another study [41], and they were found to associate with increasing depressive symptoms and perceived disability.

#### Pain acceptance

Chronic Pain Acceptance Questionnaire (CPAQ) [42] measures acceptance of chronic pain with two dimensions. The “Activities engagement”-dimension refers to an attitude of keeping up with regular life activities despite the presence of pain. The “Pain willingness” dimension suggests that the patient considers avoiding or controlling as ineffective strategies to handle pain.

CPAQ consists of 20 items that the subject answers on a 7-point scale ranging from “never true” to “always true”. Items on the pain willingness scale are reverse-scored so that increasing scores on both scales mean increasing acceptance. Psychometric studies have reproduced the measurement’s factor structure [43] and found internal consistencies to be good for the scales (α = .89 for activities engagement and α = .83 for pain willingness) [44].

### Statistical analysis

**Fig 1:**
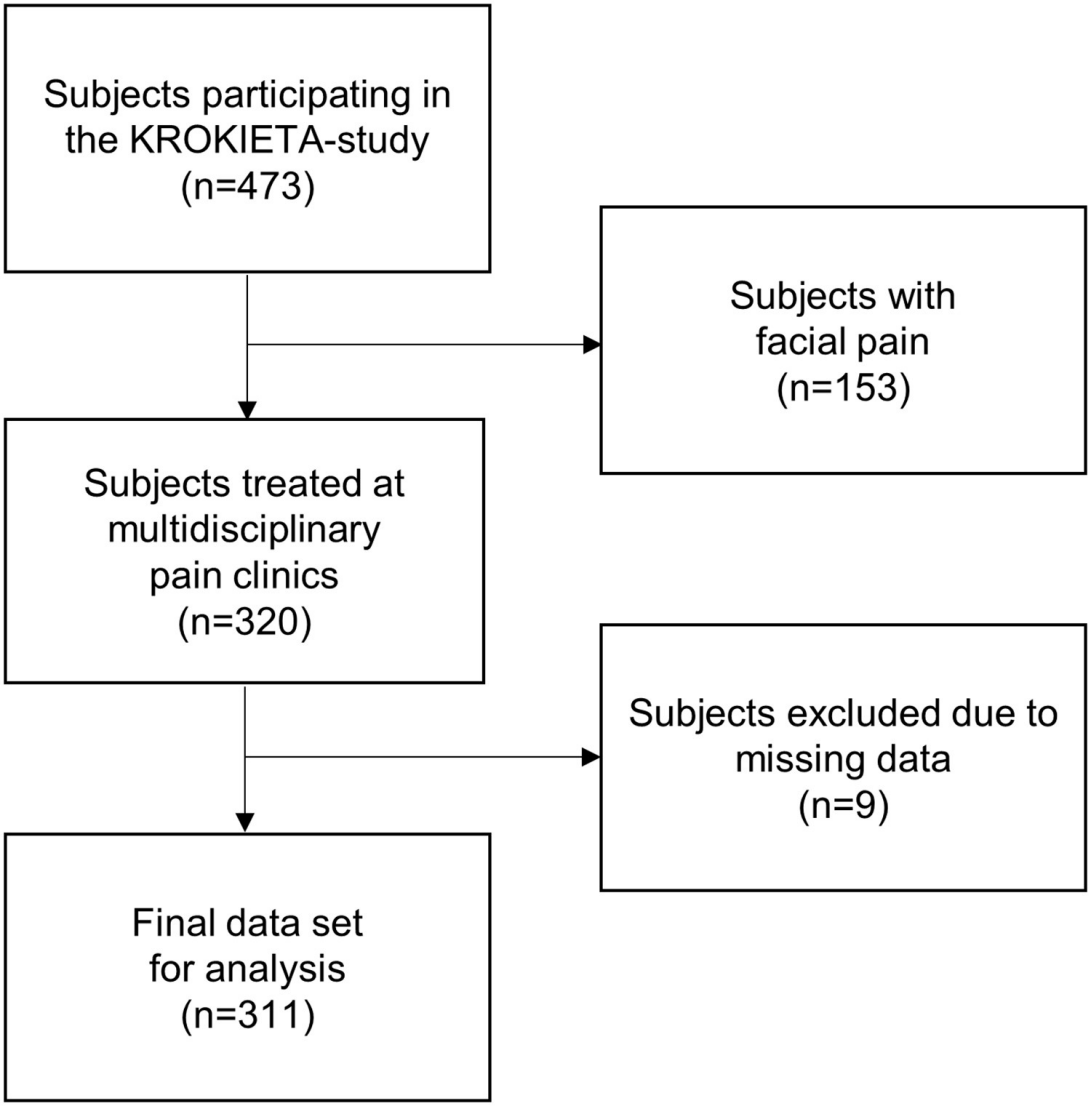
Formation of the study data set. Approximately half of the invited patients declined to participate in the study.

Data analysis started from screening of missing data on BPI. Nine subjects had completely missing data on interference items. They were excluded from subsequent analysis, leaving 311 subjects for the final data set, as shown in Fig 1. One subject had three missing items and fourteen subjects had one missing interference item, and these items were imputed by mean imputation.

Subjects were then assigned a low/high status on both activity and affective interference dimensions using 7.00 as the cut-off. The analysis continued by inspecting the formed groups against demographic variables and variables affecting physical well-being, mood, and pain intensity. Group differences were investigated through a series of one-way analysis of variances (ANOVA) and chi-square test with post hoc comparisons using Hommel’s correction.

Then, to determine whether the four groups differed on the various pain anxiety and pain acceptance dimensions, two multivariate analyses of variances (MANOVA) were conducted, first with PASS-20 subscales and then with CPAQ subscales as the dependent variables. To aid comparing groups across PASS-20 and CPAQ measurements, the raw sum scores on the subscales were converted to z-standard scores. Due to missing data on PASS-20 and CPAQ measures, there were 285 subjects in the data set for these analyses. Post hoc comparisons for group differences were conducted with a series of ANOVAs with Tukey’s Honestly Significant Difference (HSD) test. The bootstrap method was used when the theoretical distribution of the test statistics was unknown or in case of violation of the assumptions (e.g. non-normality). The effects of age, gender, and education were controlled in all analyses. The Stata 15.1, StataCorp LP (College Station, TX, USA) statistical package was used for the analyses.

## Results

### Patient demographics

The mean age of the subjects (64.4% women) was 46.9 years (SD 13.4). Most of the patients were married or co-habiting (62.3%), and the rest were either single (23.8%), divorced (10.9%), or widowed (2.2%). For most subjects, the highest educational level was either vocational or high school (65.5%) or higher education (20.4%). Slightly over half were in the work force, one in four was on a pension, and the rest were either unemployed, studying, or outside working life for other reasons. Pain had lasted between one and two years for 14.0% and over two years for 72.4% of the subjects. Of the subjects 79.3% reported pain in two or more locations. Subjects reported most often pain in the lower limbs (68.4%), low back area (57.8%), and upper limbs (46.6%) and least often pain in the stomach (19.4%), chest (14.3%), and neck areas (13.1%).

### Grouping of subjects by interference level

**Fig 2:**
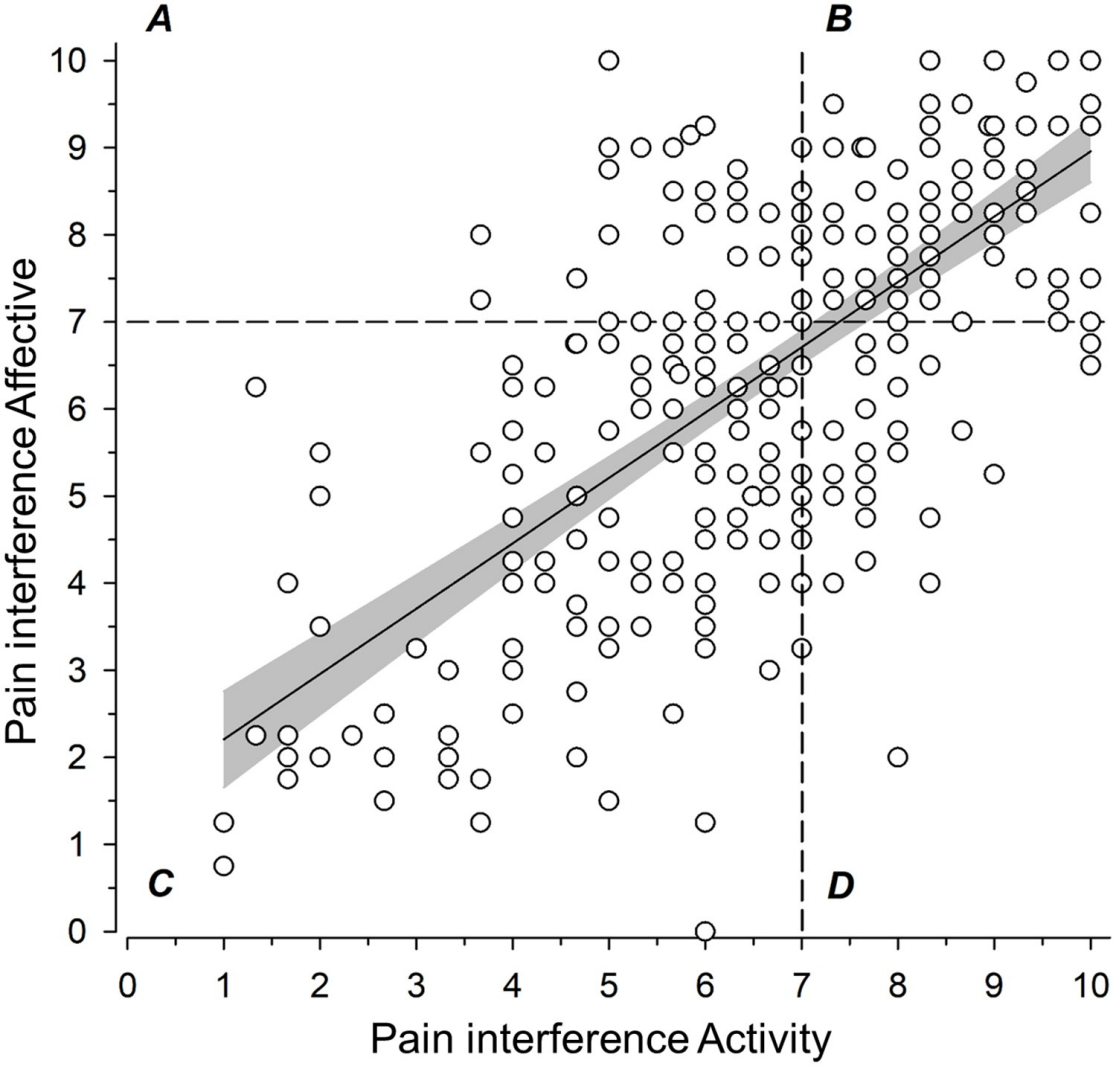
The subjects were allocated into four groups based on the activity and affective interference scores, using 7.00 as the cut-off value (0–10). A = activity low, affective high; B = activity high, affective high; C = activity low, affective low; D = activity high, affective low. Regression line with 95% confidence intervals shows the relationship between activity and affective interference (r^2^ = 0.46, 95% CI: 0.37 to 0.55).

Fig 2 shows how subjects were divided across different groups: 12% of the subjects in group A (low activity interference, high affective interference), 38% in group B (high on both interference dimensions), 38% in group C (low on both interference dimensions), and 12% in group D (high activity interference, low affective interference).

The correlation between pain intensity and activity interference was 0.63 (95% CI: 0.56 to 0.69) and between pain intensity and affective interference 0.59 (95% CI: 0.51 to 0.65).

### Group demographics, variables affecting physical well-being, and psychological resources

The groups were similar in age, gender, education, and whether pain had lasted over two years, as shown in Table 1.

**Table 1:** Demographic variables and variables affecting physical well-being, mood, and pain.

	Group				
	A Activity low Affective high n = 38	B Activity high Affective high n = 119	C Activity low Affective low n = 117	D Activity high Affective low n = 37	P-value [multiple comparison]^2^
**Demographic variables**					
Age, mean (SD)	45 (13)	47 (13)	45 (14)	46 (14)	0.64
Women, n (%)	19 (50)	78 (66)	80 (68)	28 (76)	0.10
Education years, mean (SD)	14.2 (4.8)	12.6 (2.9)	13.6 (3.3)	13.5 (3.5)	0.051
Pain duration > 2 years, n (%)	27 (71)	90 (76)	86 (74)	24 (65)	0.63
**Variables affecting physical well-being**					
BMI kg/m2, mean (SD)	27.8 (4.5)	29.9 (6.1)	27.0 (6.0)	31.0 (7.3)	<0.001 [C/B, D/C]
LTPA^1^, mean (SD)	2.3 (1.6)	1.2 (1.7)	2.4 (1.7)	1.5 (1.6)	<0.001 [A/B, A/D, C/B, D/C]
Smoking currently, n (%)	14 (37)	57 (49)	36 (31)	13 (35)	0.045 [C/B]
AUDIT total score, mean (SD)	4.1 (4.2)	3.2 (4.3)	4.2 (4.5)	4.0 (4.0)	0.45
**Mood**					
BDI-II total score, mean (SD)	17.7 (7.3)	20.3 (10.6)	11.7 (7.5)	11.4 (7.9)	<0.001 [C/A, A/D,C/B, D/B]
**Pain**					
Pain intensity, mean (SD)	6.02 (1.16)	6.88 (1.29)	4.86 (1.40)	5.95 (1.16)	<0.001 [all except A/D]

BMI = Body Mass Index, AUDIT = Alcohol Use Disorders Test, BDI-II = Beck Depression Inventory-II

^1^Leisure time physical activity; exercising sessions of ≥20 minutes per week.

^2^Count variables calculated using chi-square test and mean values by Anova. Hommel’s multiple comparison procedure was used to correct significance levels for post hoc testing (P<0.05).

Subjects with low interference on both dimensions (group C) had a mean BMI in the overweight category and they exercised on average >2 times per week, with 11% of the subjects stating that they were not able to do any exercising. The mean score in BDI showed no depressive symptomatology. In contrast, when interference was high on both dimensions (group B), the mean BMI was 2.9 kg/m^2^ higher compared with group C. The average exercising frequency was half of that of group C, and 47% reported being incapable of doing any exercise. For group B, the mean BDI score was in the moderate depressive symptom range (20–28) (all differences between C and B groups, P < 0.001).

When activity interference was low, but affective interference high (group A), the mean BMI and exercising frequency (with only 8% not exercising at all) were similar to those in group C, but the mean BDI score was significantly higher (P < 0.001) and reflected mild depressive symptomatology. Lastly, for the group with high activity interference and low affective interference (group D), the picture was reversed–the mean BMI was significantly higher, reaching the obese category, and exercising frequency was significantly lower (with 32% not exercising at all) than in group C (both P < 0.001). The mean BDI score was at the same level as that of group C, indicating no depressive symptomatology.

Smoking was less prevalent in group C, where approximately every third patient smoked, than in group B, where every other subject smoked (P = 0.045). For alcohol risk use, the AUDIT score was below the cut-off point for alcohol risk use in all groups, with no group differences in mean scores.

### Pain-related anxiety and pain acceptance

MANOVA analyses indicated a significant difference between the groups on the combined dependent variables both on PASS-20 (P < 0.001) and CPAQ (P < 0.001). ANOVAs for post hoc comparisons showed a significant difference in subscale scores between the groups (both measures, P < 0.001). Due to missing data, analyses on PASS-20 and CPAQ included 285 subjects.

**Fig 3:**
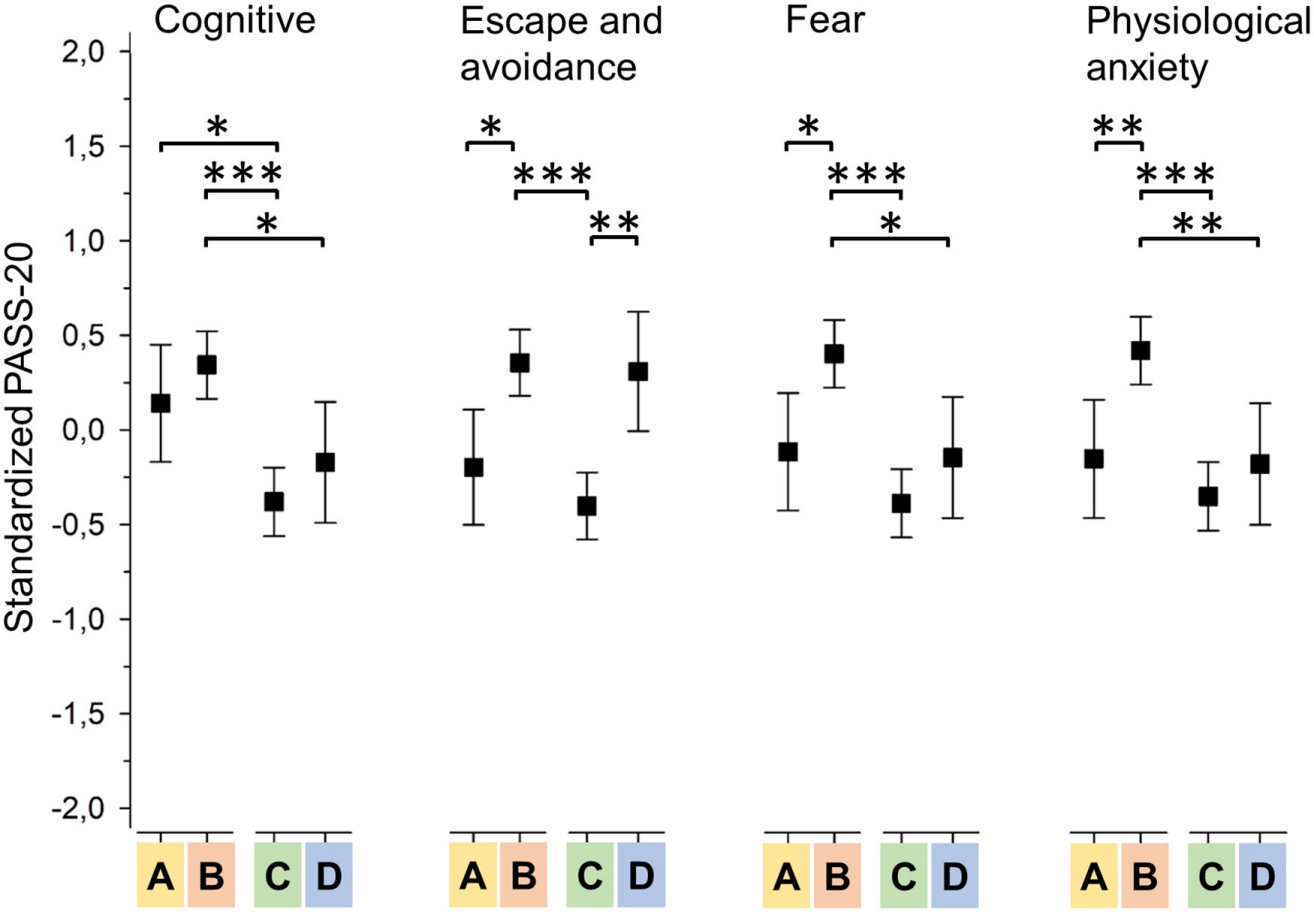
Standardized Pain Anxiety Symptoms Scale (PASS-20) subscale means with respect to different interference groups. A = activity low, affective high; B = activity high, affective high; C = activity low, affective low; D = activity high, affective low. Asterisks denote significant group differences: * = P < 0.05; ** = P < 0.01; *** = P < 0.001. Box plot whiskers represent 95% confidence intervals.

Fig 3 shows the profile of pain-related anxiety dimensions between the different pain interference groups. Group C with low activity and affective interference constantly had the lowest mean score across different anxiety dimensions, whereas group B with high activity and affective interference had the highest mean scores (C to B difference in scale scores 4.1 (95% CI: 2.7 to 5.6) for cognitive anxiety, 4.0 (95% CI: 2.6 to 5.3) for escape/avoidance, 4.6 (95% CI: 3.1 to 6.1) for fear and 4.3 (95% CI: 2.8 to 5.8) for physiological anxiety; all P < 0.001).

Group A (low activity interference, high affective interference) had a mean value that was 2.8 (95% CI: 0.7 to 4.8) scale scores higher than group C on the cognitive anxiety dimension (P = 0.02), while group D (high activity interference, low affective interference) had a mean value that was 3.7 (95% CI: 1.7 to 5.8) scale scores higher than group C on the escape/avoidance dimension (P = 0.001). Groups A and D remained at the same level as group C on fear and physiological anxiety dimensions.

The average total PASS-20 score was 36.4 (SD 17.9) for group C, 53.2 (SD 18.4) for group B, 43.2 (SD 13.0) for group A, and 43.35 (SD 18.0) for group D (P < 0.001).

**Fig 4:**
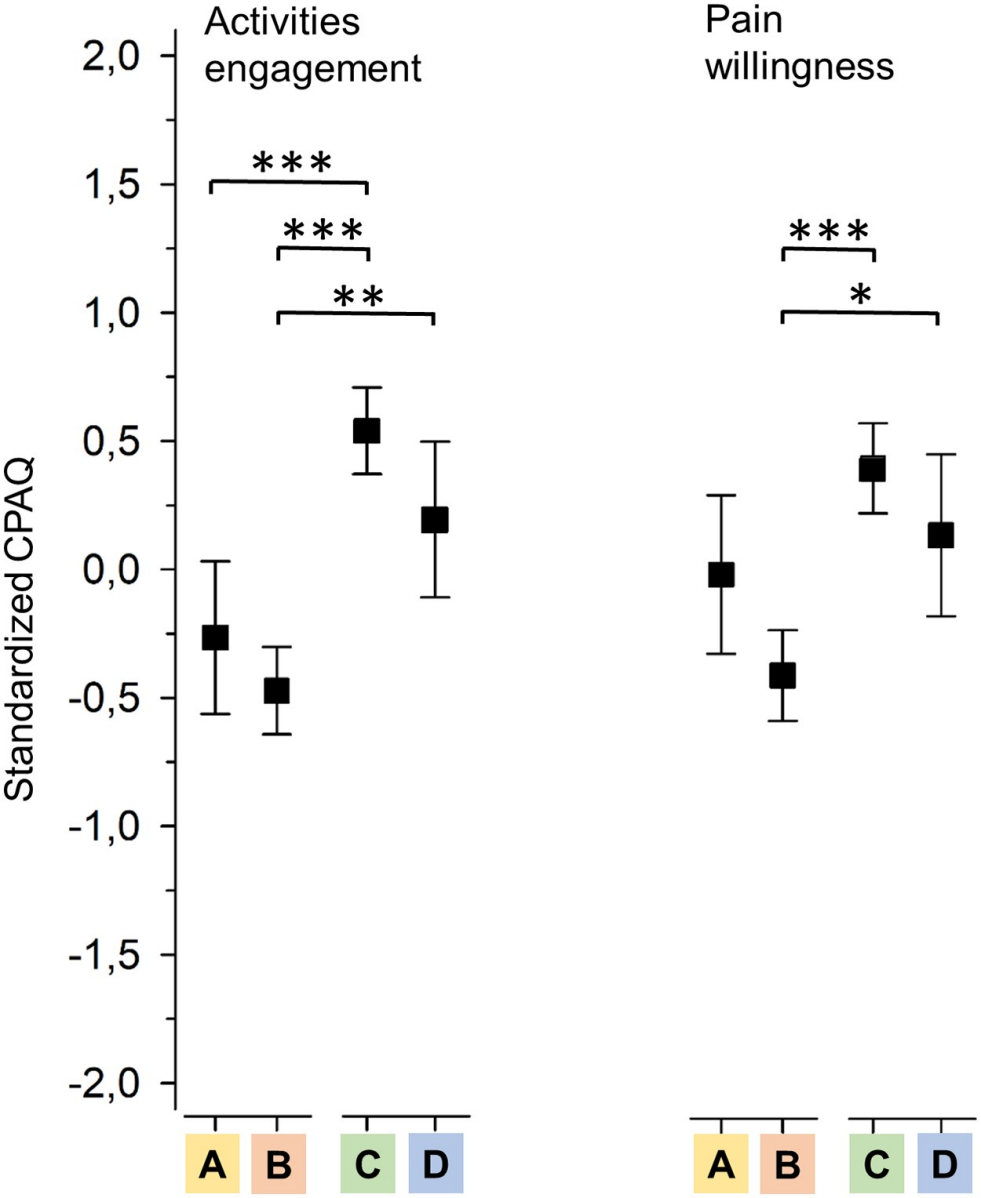
Standardized Chronic Pain Acceptance Questionnaire (CPAQ) subscale means with respect to different interference groups. A = activity low, affective high; B = activity high, affective high; C = activity low, affective low; D = activity high, affective low. Asterisks denote significant group differences: * = P < 0.05; ** = P < 0.01; *** = P < 0.001. Box plot whiskers represent 95% confidence intervals.

Groups C and B had a similar pattern for pain acceptance as for pain anxiety, as shown in Fig 4. Group C showed more and B less acceptance on both dimensions; the difference between the groups was 12.7 (95% CI: 9.6 to 15.7) scale scores for activities engagement and 6.5 (95% CI: 4.4 to 8.5) scale scores for pain willingness (both P < 0.001). Group A had a mean value of 9.7 (95% CI: 5.5 to 13.8) scale scores lower for activities engagement than group C (P < 0.001), but for pain willingness the difference was less and not significant (P = 0.10). Group D remained at the same level as group C on both pain acceptance dimensions.

## Discussion

This study grouped pain patients starting multidisciplinary pain treatment based on the type and level of reported pain interference. There were two main findings. First, adverse changes appeared in different sets of variables when pain interfered highly with either the activity dimension (i.e. walking, work, general activity) or the affective dimension (i.e. mood, relation with others, enjoyment of life, sleep). Second, when interference was high on both dimensions, adverse changes accumulated within variables affecting both physical well-being and psychological resources. There was also more pain-related fear and physiological anxiety compared to when pain interfered highly with only one of the two dimensions. The findings may help in deciding what components of multidisciplinary assessment and treatment individual patients need.

Overall, 38% of the subjects fell into the group that scored low on both activity and affective pain interference (group C). Being slightly overweight and exercising slightly over two times per week, they represented Finns as a whole [45]. Every third subject smoked in this group, this being more than the recent national average [46]. As for psychological resources, they on average appeared to have normal mood. On all pain anxiety and pain acceptance dimensions, this group was at the more favorable end, indicating that they had the most beneficial relation to pain compared with the other groups.

Pain interference was high on either of the interference dimensions for 24% of the subjects. When interference was high on the affective dimension (group A), adverse changes were seen in mood and cognitive anxiety symptoms, as expected [4], but also in activities engagement. As affective interference deals with social relations and enjoyment of life, it may not be surprising that it would be associated with reduced activities engagement, which reflects losing meaningful activities.

On the other hand, if interference was high on the activity dimension (group D), significant changes emerged, such as less exercise, as expected [6], but also weight gain and more avoidance tendencies were seen. Reduced physical activity has been associated with weight gain among pain patients [47]. The finding here on the role of avoidance suggests that it may be a key process to target in treatment planning. The fact that the average level of depressive symptoms was normal in this group may increase the probability to benefit from, for instance, participation in an exercise group targeted to pain patients [48].

A total of 38% of the patients scored high on both dimensions of interference (group B) with an associated set of adverse scores in the areas encompassed in the two previous groups (A and D). Rather worryingly, almost every other subject in group B reported being unable to participate in any exercise. Additionally, every other subject in group B smoked. Compared with the three other groups, fear and physiological anxiety were also elevated in group B patients. The mean total pain anxiety was in the “moderate” range, if using the classification by Brede et al. [41], and regarding pain acceptance this group had the worst mean scores. On the indices investigated in this study, group B patients appeared to be clearly more disadvantaged for retaining their physical well-being, having less psychological resources and experiencing more negative emotions related to pain than patients in the other groups.

The pronounced negative cognitive-emotional reactions to pain in group B are an interesting finding. Previous studies have underscored the role of catastrophizing in pain interference [49,50], and Karoly et al. [6] have elaborated on three different mechanisms. First, catastrophizing may steer more attention to interruptions in activities. Second, helplessness, which is inherent to catastrophizing, may cause withdrawal from activities. Third, repetitive thought patterns tax cognitive resources, causing interruptions to cognitively demanding activities. Similarly, low pain acceptance is connected to both limited attentional resources and restricted behavior [51]. These, together with the finding of comorbid moderate depressive symptomatology bringing further emotional load, suggest that negative emotions could be an especially important treatment target in this group [52].

The findings of our study on the different levels of complexity regarding patients’ problems (e.g. distress, pain-related anxiety) between the groups echo those of previous research categorizing pain patients in order to decide who requires more multidisciplinary components in the treatment. In the STarT Back Tool, a questionnaire categorizing back pain patients in primary care to low, medium, and high risk for pain chronification, psychological distress and pain catastrophizing are the critical indicators for high risk [53]. Low-risk patients are seen to mostly manage with informational intervention only, while high-risk patients need psycho-physical training and or a referral to a psychologist [54]. Research on the Multidimensional Pain Inventory (MPI) has produced three categories: adaptive copers (low levels of pain, functional limitations, and emotional distress), interpersonally distressed (low social support along with high pain, pain interference, emotional distress, and functional limitations), and dysfunctional patients (high levels of pain, pain interference, emotional distress, and functional limitations) [55]. Directing adaptive copers to medically oriented treatment programs, interpersonally distressed patients to cognitive-behavioral programs, and dysfunctional patients to operant group programs resulted in fewer treatment dropouts [56].

Surprisingly, no differences emerged in alcohol risk use between the groups. In prospective studies, higher pain interference has been associated with increased risk for alcohol dependence, especially in men [8,13]. Gender distribution in this study might explain the finding in part, but a more likely explanation is that prospective studies have been population-based; those patients using alcohol as a way to cope with pain may not seek treatment at all or are not referred to specialized care.

A limitation must be noted when considering the findings of this study. Approximately half of those invited to the study declined participation, which may affect the generalizability of results. However, we performed a comparison with a previous study conducted at one of the clinics participating also in this study and collecting data on all the patients entering treatment [57]. This comparison revealed that the gender distribution and the proportion of middle-aged subjects were similar between the two studies, but there were fewer older subjects and those with less than nine years of education in the current study sample. Our study did not record the demographics of those who declined to participate. Thus, we cannot explain the lower representation of elderly patients in this cohort. Older subjects may report less anxiety with regard to multidimensional pain measures [58]. Had there been more older patients in our study, the overall reported pain anxiety level might have been lower. A group possibly declining participation may comprise highly distressed individuals. For this group, the study protocol, including several questionnaires and making a laboratory visit, may have been perceived as demanding. If this is the case and had they participated, more subjects would probably have been in the group with the most problems. Nevertheless, considering the adequate sample size, equal gender distribution, and the largest age group being well represented, it is reasonable to conclude that the data allow inferences to be made about the typical population of patients entering multidisciplinary treatment.

### Clinical implications

The results of this study may benefit the assessment process of chronic pain patients entering multidisciplinary treatment. The findings indicate that if the patient presents with low interference on both activity and affective interference dimensions, the probability of benefitting from the treatment is likely to be good: less weight problems, regular exercising, better mood and the most helpful psychological reactions to pain among this patient population. This may allow proceeding in the assessment with only physician consultations. If only affective interference is high, then assessment may benefit from a more in-depth inquiry into mood, maladaptive thought patterns about pain, and lost life-fulfilling activities, e.g. a consultation with a psychologist. When only activity interference is high, inquiry should focus more on issues of weight gain, exercise, and pain avoidance behavior. The patient might benefit from seeing a physiotherapist as part of the assessment. Finally, when the patient presents with high interference on both dimensions, the risk for reduced physical and psychological well-being have likely accumulated, and the psychological reactions to pain seem highly important targets in the assessment. For these patients, the findings support the need for full multidisciplinary assessment.

### Implications for future research

Future research should seek whether the pain interference categories identified in this study and their relationships to the studied variables can be replicated. A longitudinal study would provide an opportunity to investigate whether the four categories differ in treatment pathways and outcomes. Another interesting question would be to find out whether the introduction of the pain interference categories could streamline the allocation of chronic pain patients into appropriate components of multidisciplinary assessment and management.
